# Potential Biomarkers for Predicting Depression in Diabetes Mellitus

**DOI:** 10.3389/fpsyt.2021.731220

**Published:** 2021-11-29

**Authors:** Xiuli Song, Qiang Zheng, Rui Zhang, Miye Wang, Wei Deng, Qiang Wang, Wanjun Guo, Tao Li, Xiaohong Ma

**Affiliations:** ^1^Clinical Psychology, Yantai Affiliated Hospital of Binzhou Medical University, Yantai, China; ^2^Psychiatric Laboratory and Department of Psychiatry, West China Hospital, Sichuan University, Chengdu, China; ^3^School of Computer and Control Engineering, Yantai University, Yantai, China; ^4^Information Center, West China Hospital, Sichuan University, Chengdu, China

**Keywords:** diabetes mellitus, depression, support vector machine, biomarkers, machine learning method

## Abstract

**Objective:** To identify the potential biomarkers for predicting depression in diabetes mellitus using support vector machine to analyze routine biochemical tests and vital signs between two groups: subjects with both diabetes mellitus and depression, and subjects with diabetes mellitus alone.

**Methods:** Electronic medical records upon admission and biochemical tests and vital signs of 135 patients with both diabetes mellitus and depression and 187 patients with diabetes mellitus alone were identified for this retrospective study. After matching on factors of age and sex, the two groups (*n* = 72 for each group) were classified by the recursive feature elimination-based support vector machine, of which, the training data, validation data, and testing data were split for ranking the parameters, determine the optimal parameters, and assess classification performance. The biomarkers were identified by 10-fold cross validation.

**Results:** The experimental results identified 8 predictive biomarkers with classification accuracy of 78%. The 8 biomarkers are magnesium, cholesterol, AST/ALT, percentage of monocytes, bilirubin indirect, triglyceride, lactic dehydrogenase, and diastolic blood pressure. Receiver operating characteristic curve analysis was also adopted with area under the curve being 0.72.

**Conclusions:** Some biochemical parameters may be potential biomarkers to predict depression among the subjects with diabetes mellitus.

## Introduction

Diabetes mellitus is a chronic illness affecting about 347 million people worldwide in 2017, and this number is expected to increase more than half by 2035 (1, 2). The disease will also lead to emotional distress other than physical symptoms and impose psychosocial impacts on life quality, which complicates its management.

Depression and diabetes mellitus are common comorbid conditions (3). A meta-analysis reported that patients with diabetes mellitus more than doubled the odds of developing depression (3). Another study described that depression was highly prevalent, affecting ~26% of the patients with diabetes mellitus (4). In addition, depression was found to be associated with a greater number of complications of diabetes mellitus (5). Furthermore, depression itself is a disabling disease and imposes a significant impact on life quality by undermining physical health (6) and impairing cognitive functions (7). Therefore, it is not surprising that diabetes mellitus comorbidity with depression is associated with higher morbidity and mortality rates, decreased compliance with treatment, poorer functionality, poor glycemic control, and more expenditure on use to health services (7–12). A prospective study involving more than 4,000 patients having diabetes mellitus with comorbidity of depression reported a higher risk of developing macrovascular complications, even when variables such as the type of treatment and the existed history of complications before the study were controlled (13). This highlights the severity of diabetes mellitus in comorbidity with depression and the need to treat both conditions concurrently.

Comorbid depression in diabetes mellitus might be considered not as the result of mental problem only, but more important, as an early sign of a multi-systemic disorder. Thus, medical monitoring is an important component of case assessment. The diagnosis of depression mainly depends on doctors' clinical experience and scale. The lack of objective indicators, the strong subjective consciousness of doctors and patients, and the avoidance or denial in some symptoms due to patients' insufficient understanding of the disease interfere with the accuracy of scale score; and this may affect the correct diagnosis of the disease (14–16). Therefore, it is particularly important to identify objective indicators of depression diagnosis and establish scientific diagnostic methods. Nonetheless, very few approaches have been proposed to facilitate early prediction depression in patients having diabetes mellitus because objective indicators of laboratory examinations are rare.

Recently, machine learning algorithms have been widely used in the medical sciences. It was reported that machine learning algorithms in combination with smartphone-based data will be a new approach to classify affective states accurately in bipolar disorder (17). In addition, machine learning methods may be used to predict treatment effect of electroconvulsive therapy (ECT) (18), cognitive behavioral therapy (CBT) (19), and clozapine (20); or to help diagnostic clarification (21). According to Kim et al., comprehensive machine-learning methods that adopt supervised classification and appropriate feature selection methods that have interaction with the classifier show particular advantages in predicting complicated disorders with multi-facet etiology such as depression (22). Support Vector Machine (SVM) is a method of machine learning and is of great significance in accurately identifying depression among patients with diabetes mellitus in clinical practice. This method provides insights for understanding the underlying pathological mechanisms of depression.

Previous studies have reported a high accuracy of over 80% in differentiating patients with depression from healthy controls, using machine learning methods to analyze heart rate variability (HRV) and/or protein markers (22, 23). Nevertheless, the existing extraction procedures of parameters are usually complex. For example, Kuang et al. (23) need to examine the 64 features of HRV in the Ewing test including the different states—resting, valsalva, deep breathing, and standing states. By contrast, our study was much simpler in that only easy-to-obtain routine biochemical tests and vital signs of patients were needed. By SVM, the best executing classification system can be set up with a small number of parameters that are selected from a variety of biochemical tests and vital signs.

To address this need, we proposed using SVM to identify potential prediction biomarkers for depression in patients with diabetes mellitus.

## Materials and Methods

### Data Acquisition

Biochemical tests and vital signs were obtained from electronic medical records of admissions in West China Hospital of Sichuan University between January 1, 2011 and October 31, 2016. A total of 322 patients were divided into two groups: 135 with both diabetes mellitus and depression (comorbidity group), and 187 with diabetes mellitus alone (DM group). Specifically, the DM group was diagnosed using the ICD - 10 categories E10.x - E14.x, and the depression in comorbidity group was diagnosed using the ICD - 10 categories F32.x and F33.x. To avoid confounding, patients with other diseases or of non-Han ethnicities were excluded. Each department had different biochemical parameters checked as appropriate, and we analyzed the same biochemical parameters for both groups (Table 1). Written informed consent had been obtained from all patients, and the Institutional Ethics Committee of Sichuan University approved this study.

**Table 1:** The 52 biochemical tests and 5 vital signs.

**52 biochemical tests and 5 vital signs**							
1	Red blood count (RBC)	14	Acidophil absolute value	27	High-density lipoprotein cholesterol (HDL-C)	40	Glutamyl transpeptidase
2	Hemoglobin (HGB)	15	Basophilic cell absolute value	28	Low-density lipoprotein cholesterol (LDL-C)	41	Blood urea nitrogen
3	Mean cell hemoglobin concentration (MCHC)	16	Creatine kinase (CK)	29	Total protein	42	Sodium
4	Platelet count (PLT)	17	Lactic dehydrogenase (LDH)	30	Albumin (A)	43	Potassium
5	White blood cell count (WBC)	18	Total bilirubin	31	Globulin (G)	44	Chlorine
6	Percentage of neutrophils	19	Direct bilirubin	32	A/G	45	Anion gap
7	Percentage of lymphocytes	20	Bilirubin indirect	33	Creatinine	46	Serum cyscatin-c
8	Percentage of monocytes	21	Hydroxybutyrate dehydrogenase	34	Uric acid	47	Hydroxybutyric acid
9	Eosinophil percentage	22	Triglyceride	35	Aspartate aminotransferase (AST)	48	Urine RBC
10	Basophil percentage	23	Cholesterol	36	Alanine aminotransferase (ALT)	49	Urine WBC
11	Absolute value of neutrophils	24	Calcium	37	AST/ALT	50	Urine conductivity
12	Absolute value of the lymphocyte	25	Magnesium	38	Alkaline phosphatase (ALP)	51	Urine specific gravity
13	Absolute value of the monocytes	26	Phosphorus	39	Glucose	52	Urine potential of hydrogen (U-PH)
1	Body temperature	3	Respiration	5	Diastolic blood pressure		
2	Pulse	4	Systolic blood pressure				

### Data Processing

**Figure 1:**
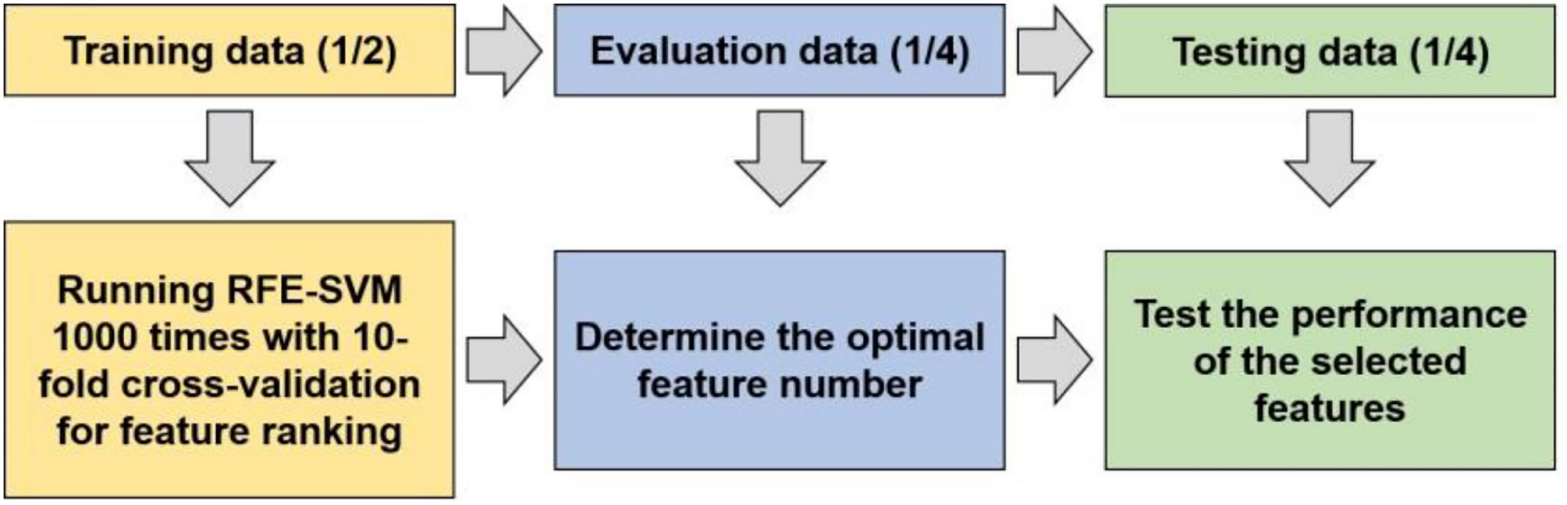
The flowchart of data processing.

To detect whether biochemical tests and vital signs can function as markers for predicting depression in diabetes mellitus, a RFE-SVM algorithm was adopted to identify the markers and assess the classification performance (Figure 1).

Before applying the machine learning method to identify predictive markers, propensity score matching (PSM) analysis was performed because age and sex differed significantly between the DM and comorbidity groups. After the matching analysis, the experimental data were split into training data, validation data, and testing data with the proportion of 1/2, 1/4, 1/4 to obtain feature ranking, determine the optimal features, and assess the classification performance. Specifically, the implementation of the machine learning can be summarized as follows:

① Determine the feature ranking by recursive feature elimination-based SVM on the training data. The experiments were repeated 1,000 times with 10-fold cross validation.

**Figure 2:**
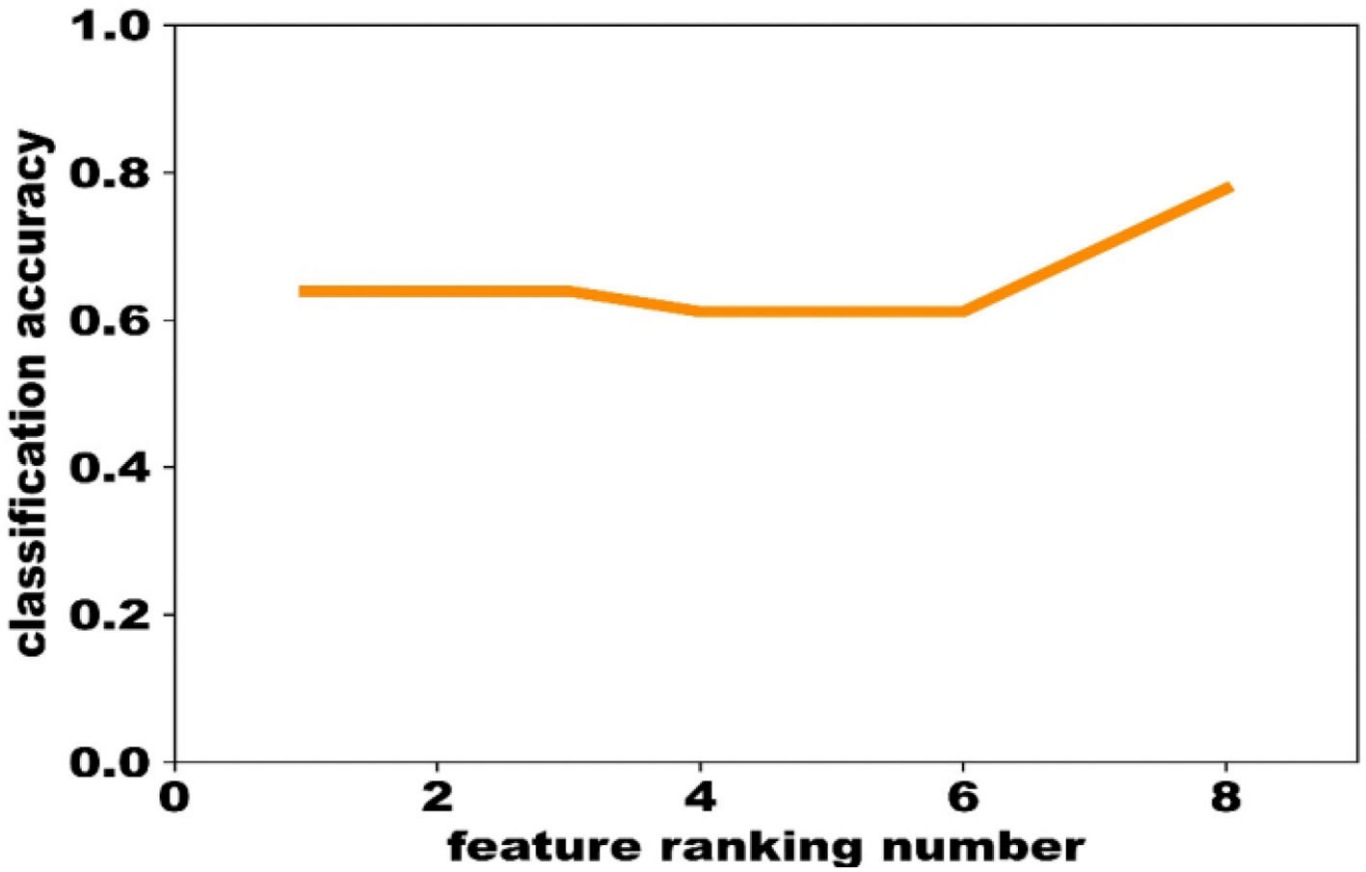
The procedure of feature selection on the evaluation data.

② Train a SVM classification model on the training data using the liblinear toolbox, and determine the most predictive features using the evaluation data based on the feature ranking obtained above. The feature that ranked No. 1 was first used to optimize the model, and the performance was evaluated by the validation data. Then, the feature that ranked No. 2 was combined to optimize the model and to compare the performance with the previous one. If the performance of the latter classifier was worse than the former, the feature that ranked No. 2 would be removed. In this way, only the features that could increase the classification accuracy were remained, and finally we obtained 8 biomarkers (Figure 2).

③ Train the classification model on the training data with the selected 8 biomarkers, and assess the performance on the testing data by the measurements of accuracy, AUC, sensitivity, and specificity.

### Statistical Analysis

Statistical analysis was performed using SPSS 20.0. Two-sample *t*-test and chi-squared test were used for comparison between groups. Propensity score matching (PSM) analysis was performed for matching age and sex. Statistical significance was set at *P* < 0.05 for both tests.

## Results

Table 1 showed analyzed 52 biochemical tests and 5 vital signs for both groups, including red blood count (RBC), acidophil absolute value, high-density lipoprotein cholesterol (HDL-C), glutamyl transpeptidase, hemoglobin (HGB), basophilic cell absolute value, low-density lipoprotein cholesterol (LDL-C), blood urea nitrogen, mean cell hemoglobin concentration (MCHC), creatine kinase (CK), total protein, sodium, platelet count (PLT), lactic dehydrogenase (LDH), albumin (A), potassium, white blood cell count (WBC), total bilirubin, globulin (G), chlorine, percentage of neutrophils, direct bilirubin, A/G, anion gap, percentage of lymphocytes, bilirubin indirect, creatinine, serum cyscatin-c, percentage of monocytes, hydroxybutyrate dehydrogenase, uric acid, hydroxybutyric acid, eosinophil percentage, triglyceride, aspartate aminotransferase (AST), urine RBC, basophil percentage, cholesterol, alanine aminotransferase (ALT), urine WBC, absolute value of neutrophils, calcium, AST/ALT, urine conductivity, absolute value of the lymphocyte, magnesium, alkaline phosphatase (ALP), urine specific gravity, absolute value of the monocytes, phosphorus, glucose, urine potential of hydrogen (U-PH), body temperature, respiration, diastolic blood pressure, Pulse, and systolic blood pressure.

In this retrospective study, medical records upon admission of 322 patients were selected. After the matching analysis, there are 72 samples in the DM group and the comorbidity group, respectively. Demographic characteristics of the DM group (*n* = 72) and the comorbidity group (*n* = 72, F32.x: 106 and F33.x: 29) were summarized (Table 2). The mean (SD) age of subjects was 56.13 (7.98) years in the DM group and 54.93 (7.62) years in the comorbidity group. There were not different between two groups on age and sex (male: 31, respectively). Eight features were computed in 10-fold cross-validation experiments, repeated 1,000 times with SVM, including magnesium, cholesterol, AST/ALT, percentage of monocytes, bilirubin indirect, triglyceride, lactic dehydrogenase (LDH), and diastolic blood pressure (Table 2).

**Table 2:** Demographics and biomarkers of experimental results of 144 diabetes mellitus patients with and without depression.

	**DM group (*n* = 72)**	**Comorbidity group (*n* = 72)**	**Statistics**	***P***
Sex	Male (*n* = 31)	Male (*n* = 31)	0.00	1.00
Age	56.13 ± 7.98	54.93 ± 7.62	0.92	0.36
Magnesium	0.84 ± 0.08	0.88 ± 0.12	−2.86	0.005
Cholesterol	4.25 ± 0.57	4.71 ± 0.95	−3.57	<0.001
AST/ALT	1.03 ± 0.38	0.97 ± 0.36	1.00	0.32
Percentage of monocytes	5.38 ± 1.58	5.80 ± 1.46	−1.65	0.10
Bilirubin indirect	7.31 ± 2.94	8.54 ± 4.59	−1.92	0.06
Triglyceride	1.40 ± 0.62	1.82 ± 1.43	−2.30	0.02
lactic dehydrogenase (LDH)	165.63 ± 27.78	155.07 ± 47.87	1.62	0.11
Diastolic blood pressure	77.81 ± 10.77	78.87 ± 9.32	−0.63	0.53

**Figure 3:**
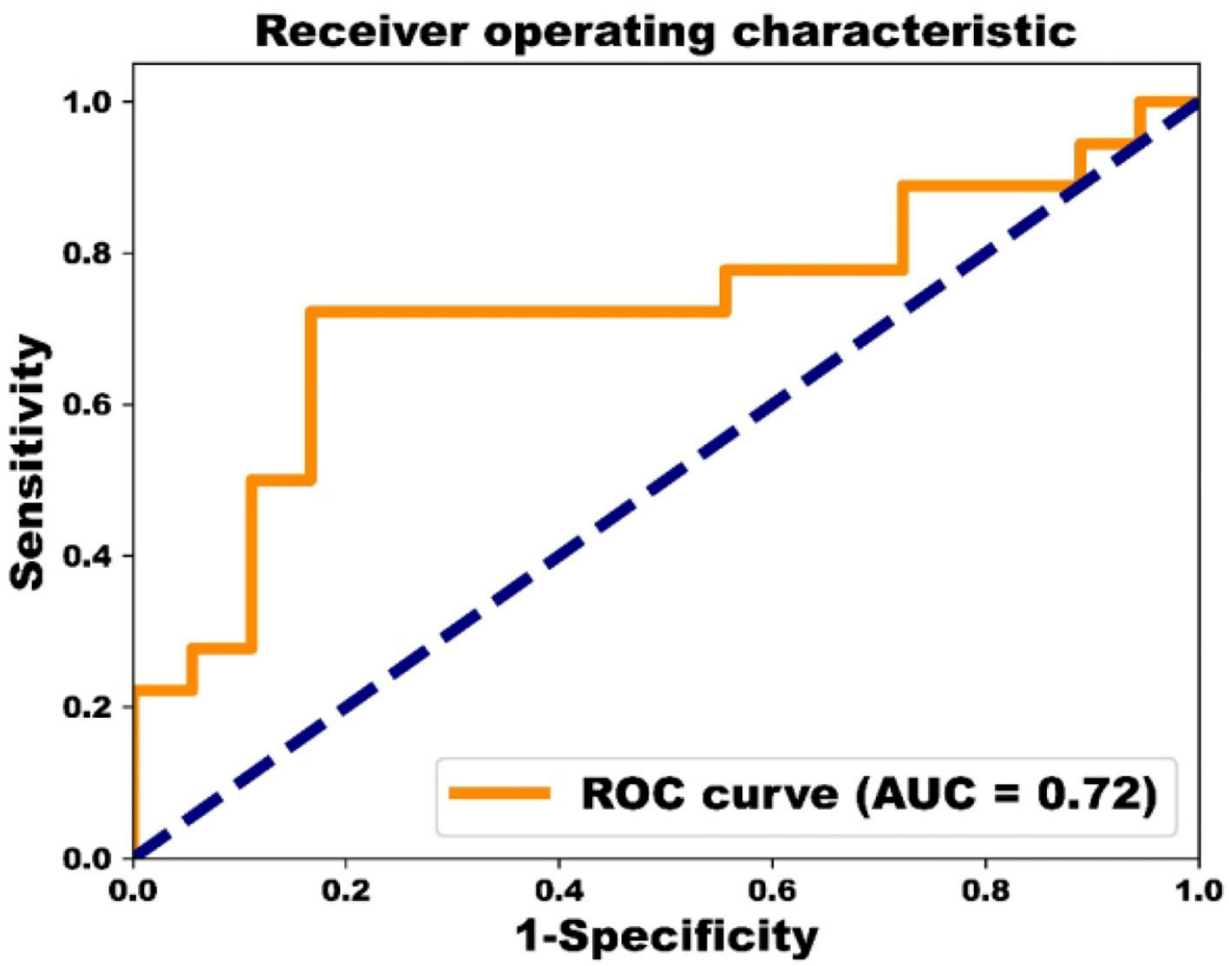
ROC curve analysis with AUC value.

The performance of classification of both groups reached 83% for sensitivity, 72% for specificity, 78% for accuracy, and 0.72 for AUC based on ROC analysis (Figure 3).

## Discussion

In this retrospective study, we found 8 important depression biomarkers using SVM. These biomarkers are magnesium, cholesterol, AST/ALT, percentage of monocytes, bilirubin indirect, triglyceride, lactic dehydrogenase, and diastolic blood pressure, which differentiate depression in patients with diabetes mellitus at an overall classification accuracy of 78%. Eight identified factors imply that modulation of the inflammatory, immune, energy metabolism, and lipid metabolism pathways were mainly involved in the pathophysiology of depression in patients with diabetes mellitus.

We found four biomarkers involved in inflammatory and immune pathway including magnesium, AST/ALT, percentage of monocytes, and bilirubin indirect. Depression often comorbid with diabetes, metabolic disorders and other diseases, and is associated with inflammatory and oxidative stress (24). Type 2 diabetes usually begins with insulin resistance, and a relationship between depression and insulin resistance also exists (25). Diabetes can cause a rise in blood sugar and insulin levels and has an effect on inflammation that may contribute to depression. Recent studies have shown that oxidative stress may enhance induction of HO-1 expression, which may result in insulin resistance and insufficiency (26, 27). It is clear that increased oxidative stress may lead to insulin resistance and impose an impact on insulin secretion in patients having depressive disorder (27). One study demonstrated that reducing inflammation through non-drug treatments such as psychological interventions, physical exercises, and meditation can play a role in preventing depression (28). Magnesium has received great concern over its potential role in the pathophysiology of depression (29–31). Many studies support the hypothesis that inflammatory cytokines are important factors in the pathogenesis of MDD (32). Auffray et al. suggested that monocytes mediate fundamental regulatory and effector functions in immune inflammatory responses (33). Previous study have indicated that MDD patients with elevated serum TNF-α and IL-1β levels display marked alterations in circulating monocytes and exhibit a systemic proinflammatory state compared to healthy controls (34). Additionally, studies have shown that the percentage of monocytes decreased by imipramine treatment can be enhanced by stress exposure (35). New evidence shows that antidepressant treatment can reduce inflammation and improve mitochondrial dysfunction in patients with depression (36, 37). Also, studies have indicated that increased ALT levels were an independent predictor of depression onset (38).

We also found one biomarker potentially related to energy metabolism. The biomarker is lactic dehydrogenase. It is responsible for the conversion of lactic acid to pyruvic acid, an important step in the production of cellular energy (39). Kato et al. found that healthy nurses' depressive symptoms shown on CES-D under the stressful conditions were significantly negatively correlations with lactate dehydrogenase activities (*r* = −0.29, *p* = 0.0065) (40). We observed that patients with both diabetes mellitus and depression had lower concentrations of lactic dehydrogenase compared to those with diabetes alone. In another study, Ivana Perić et al. showed an increase in lactate dehydrogenase (LDH) levels after Tianeptine treatment in stressed rats (41). After antidepressant treatment, LDH level is increased and depression was alleviated, suggesting that LDH may be related to the pathological basis of depression (41). Additionally, we found some other biomarkers that may be related to lipid metabolism, including cholesterol and triglyceride. Clinical and experimental evidence has suggested that plasma lipids might be an important factor in the pathophysiological mechanisms related to depression (42). Higher level of cholesterol was observed in patients with depression than in controls (27, 43). In agreement with this finding, increased levels of cholesterol were found to be associated with comorbidity of diabetes mellitus and depression in our study. A recent study has analyzed 230 metabolic markers and reported a clear and unique profile of circulating lipid metabolites related to depression (44). Bot et al. has found that depression is associated with higher triglyceride (44), which is consistent with our results in this study. A previous study has shown that activation of the proinflammatory response results in a decrease in HDL cholesterol and phospholipids, as well as an increase in TG mediated by compensatory production and accumulation of phospholipid-rich VLDL (45).

Depression is common in patients with diabetes, and there is a bidirectional association between diabetes and depression. Many mechanisms are considered to be involved in the link between depression and diabetes, including HPA axis dysregulation, immune and inflammatory mechanisms, brain insulin resistance, circadian rhythm dysregulation, shared genetic factors and more (46). For example, the immune system has also been implicated in the co-occurrence of depression and diabetes. Monocytes in the peripheral blood are the most important cells in the innate system, which produce cytokines involved in the development of inflammation in patients with diabetes (47). Previous study have shown that imbalances in Mg^2+^ status can increase insulin resistance, inhibit translocation of glucose transporter type 4, induce oxidative stress, affect lipid metabolism, and impair the antioxidant system of endothelial cells, thus promoting the progression of DM (48). Additionally, lactate metabolic pathways are important for understanding the pathogenesis of diabetes. It has been reported that pyruvate is reduced to lactate in the cytoplasm by lactate dehydrogenase without oxygen consumption, and excess lactate is generated in diabetes (49). Hildrum et al. found that patients with anxiety and depression had higher diastolic blood pressure at 11-year follow-up in these populations, but presented lower diastolic blood pressure at 22-year follow-up, which may be related to antidepressants (50). Trento found that self-management education improved blood pressure in patients with type 2 diabetes (51). These factors are also present in patients with depression.

Changes in triglyceride, AST, ALT, bilirubin indirect, lactic dehydrogenase, and cholesterol etc. in blood are not specific to depression and may be present in other psychiatric disorders such as eating disorders (52), schizophrenia (53, 54), and bipolar disorder (55, 56). Researchers suggested that a single biomarker often lacks in sensitivity and specificity (27) and thus may not well-distinguish depression from other diseases. Monitoring changes in multiple factor levels will provide a more comprehensive and accurate assessment, which can help us better understand the disease status and characteristics of specific diseases. Although the model of multiple biomarkers is more conducive for the diagnosis of diseases, it is usually used in the diagnosis of cancer instead of nervous system diseases (57, 58). Our study is advantageous in that laboratory biochemical indexes are routine examinations in clinical settings, which could be obtained with minimal invasiveness, maximal convenience, and low cost, thus having a great potential for wider clinical access and more efficient population screening. Because the biochemical tests of the two groups were not identical, the different ones were deleted. The lack of biochemical tests as variables in SVM learning affected accuracy, which is one limitation of the present study. Second, the parameters chosen retrospectively instead of consecutively were inadequate and included only those that were clinically applicable. This may have caused an enrollment bias and an erroneous classification by the algorithm. This is one of the major methodological limitations of the present study, which should be remedied in future investigations using a prospective and consecutive design.

In conclusion (1) SVM can facilitate clinical diagnosis of depression in patients with diabetes mellitus using commonly available laboratory parameters. (2) Eight potential biomarkers were identified for depression diagnosis in patients with diabetes mellitus.

## Data Availability Statement

The datasets generated during and/or analyzed during the current study are available from the corresponding author on request.

## Ethics Statement

Written informed consent was obtained from the patient for the case report. The Institutional Ethics Committee of Sichuan University approved this study. The patients/participants provided their written informed consent to participate in this study. Written informed consent was obtained from the individual(s) for the publication of any potentially identifiable images or data included in this article.

## Author Contributions

XS, QZ, and XM participated in study design, data analysis, accrual of study participants, and manuscript writing and review. RZ and MW participated in data analysis and critical revisions for important intellectual content. WD, QW, WG, and TL review of manuscript. All authors have read and approved the final version of the manuscript.

## Funding

This research was partly funded by National Natural Science Foundation of China (Grant No. 81671344).

## Conflict of Interest

The authors declare that the research was conducted in the absence of any commercial or financial relationships that could be construed as a potential conflict of interest.

## Publisher's Note

All claims expressed in this article are solely those of the authors and do not necessarily represent those of their affiliated organizations, or those of the publisher, the editors and the reviewers. Any product that may be evaluated in this article, or claim that may be made by its manufacturer, is not guaranteed or endorsed by the publisher.
